# Seasonal Characterization of the Aerobiome in Hematopoietic Stem Cell Transplant Rooms: Potential Risk for Immunosuppressed Patients

**DOI:** 10.3390/microorganisms12112352

**Published:** 2024-11-18

**Authors:** Emilio Mariano Durán-Manuel, Edgar Fiscal-Baxin, Andres Emmanuel Nolasco-Rojas, Miguel Ángel Loyola-Cruz, Clemente Cruz-Cruz, Marianela Paredes-Mendoza, Adolfo López-Ornelas, Dulce Milagros Razo Blanco-Hernández, Nayeli Goreti Nieto-Velázquez, Aída Verónica Rodríguez-Tovar, Adrián Ramírez-Granillo, Enzo Vásquez-Jiménez, Verónica Fernández-Sánchez, Erika Gómez-Zamora, Mónica Alethia Cureño-Díaz, Andrea Milán-Salvatierra, Carlos Alberto Jiménez-Zamarripa, Claudia Camelia Calzada-Mendoza, Juan Manuel Bello-López

**Affiliations:** 1 Hospital Juárez de México, Mexico City 07760, Mexicoalf2228@yahoo.com.mx (V.F.-S.); erika19723@hotmail.com (E.G.-Z.); dracureno@yahoo.com.mx (M.A.C.-D.); mil_an_drea@hotmail.com (A.M.-S.); 2 Sección de Estudios de Posgrado, Escuela Superior de Medicina, Instituto Politécnico Nacional, Mexico City 11340, Mexico; carlosajz@msn.com; 3 División de Tecnología Ambiental, Universidad Tecnológica de Nezahualcóyotl, Nezahualcóyotl 57000, Mexico; 4 Hospital Nacional Homeopático, Hospitales Federales de Referencia, Mexico City 06800, Mexico; 5 Departamento de Microbiología, Escuela Nacional de Ciencias Biológicas, Instituto Politécnico Nacional, Mexico City 11340, Mexico; 6 Facultad de Estudios Superiores Iztacala, Universidad Nacional Autónoma de México, Mexico City 54090, Mexico; 7 Hospital Psiquiátrico “Dr. Samuel Ramirez Moreno”, Valle de Chalco Solidaridad 56619, Mexico

**Keywords:** aerobiome, fungi, bacteria, transplantation, hematopoietic stem cells

## Abstract

Infections pose a risk for patients undergoing hematopoietic stem cell (HSC) transplants due to their immunosuppression, making them susceptible to opportunistic infections. Therefore, understanding the composition of the aerobiome in this area is vital. The aim of this study was to characterize the aerobiome in an HSC transplant area, evaluating the impact of infrastructure and health personnel operations on air contamination. The environmental parameters and aerobiome of the HSC transplant area at Hospital Juárez de México were quantified over one year. Finally, a double-entry Vester matrix was constructed to classify problems according to their degree of causality. The abundance and taxonomic diversity of the aerobiome were dependent on seasonality, environmental factors, and high-efficiency filtration. Gram-positive bacteria predominated, followed by fungi and Gram-negative bacteria. ANOVA revealed significant differences in the bacterial aerobiome but not in the fungal aerobiome among the transplant rooms. Clinically, fungi such as *Aspergillus fumigatus*, *Alternaria* spp., *Cladosporium* spp., and *Penicillium* spp. were identified. ESKAPE bacteria typing revealed clonal dispersion. Finally, the Vester matrix highlighted critical problems associated with contamination due to the absence of HEPA filtration and non-adherence in patient management practices. HEPA filtration and positive pressure are essential to improve the air quality and reduce the microbiological load. However, the control areas will depend on patient management and routine activities, such as entry protocols in controlled areas.

## 1. Introduction

Scientific and technological advances in the field of hematopoietic stem cell (HSC) transplants have revolutionized the treatment of several oncohematological diseases, with acute lymphoblastic leukemia (ALL) being one of the most important because of its high incidence in the pediatric and juvenile population in Latin America [1,2,3]. HSC transplantation is a multidisciplinary procedure that involves the participation of various healthcare professionals, including hematologists, oncologists, geneticists, immunologists, and infectologists, among others. Therefore, the success of a transplant will depend on the synergy between each one of these disciplines; however, these procedures are inherently associated with significant risks, including liver disease, cardiovascular disease, endocrine disorders, graft-versus-host reactions, and opportunistic infections [4,5,6,7]. Regarding the latter, prior to HSC transplantation, the patient undergoes ablation and, consequently, opportunistic infections can easily occur, with bacterial sepsis, mycosis, and invasive pulmonary fungemia being the most common [8,9,10].

Even when these procedures include prophylactic antimicrobial treatment before and after transplantation, the presence of resistant microorganisms can occur and becomes more clinically relevant due to antibiotic therapy failure [11,12,13]. In this context, the presence of nosocomial antibiotic-resistant bacteria on surfaces and medical devices can lead to cross-contamination events and thus increase the length of stay, morbidity, and mortality due to infectious events.

In a previous study by our working group, we identified the clonal spread of the ESKAPE bacteria in the adult intensive care unit (ICU) for COVID-19 patients [14]. In this work, we showed and discussed the problematic and negative impact of the presence of antibiotic-resistant microbiological contamination in patients with respiratory support. In contrast, regarding the microbiological control of HSC transplantation rooms, beyond surfaces and medical devices, air becomes relevant as it is the vehicle for the dispersal of microorganisms in patients who receive a transplant [15,16,17].

The aerobiome in such rooms is a critical factor, yet it has been underestimated as an “invisible” biological entity. The air surrounding a patient who has received a transplant is a complex mixture of viable and non-viable particles; within the viable particles are microorganisms, such as fungal spores and bacteria, which can play a determining role in the acquisition of post-transplant infections [18,19,20,21]. The negative impact of non-viable particulate matter (PM), specifically PM2.5 in transplant areas, has been rarely studied, and the findings that have been generated have not shown clear evidence to support its relationship with the clinical worsening of transplant patients [22]. Nevertheless, their role as drivers of chronic degenerative diseases in other settings has already been reported [23,24].

The detailed seasonal characterization of the cultivable bacterial and fungal aerobiome and PM in transplant areas has become a key priority, as it provides information for the implementation of effective infection control strategies. The strengthening of patient safety during their hospital stays to reduce morbidity and mortality highlights the need for continuous improvement in the infrastructure of such wards. The aim of this study was the seasonal characterization of the bacterial and fungal aerobiome and PM in the HSC transplantation rooms of Hospital Juárez de México (HJM), a tertiary hospital located in the north of Mexico City. Implications for the seasonal presence of bacteria, fungi, and clones of the ESKAPE bacteria in the aerobiomes of HSC transplant rooms are analyzed and discussed.

## 2. Materials and Methods

### 2.1. General Description of the HSC Transplant Area at HJM

The HSC transplant area at HJM is a controlled area that was built on a total surface area of 138 m^2^. This area is divided into two care areas: one for HSC donors of 34 m^2^ (room A), with three beds, and another area for recipients of 38 m^2^. The remaining area is for the reception, lock chamber, bathroom, and control site. The HSC recipient area is subdivided into three single rooms of 12.71 m^2^ each (rooms B, C, and D). Room D is the only room with ten pascals of positive pressure provided by the presence of an air handler unit coupled to a high-efficiency particle arresting (HEPA) filter.

**Figure 1:**
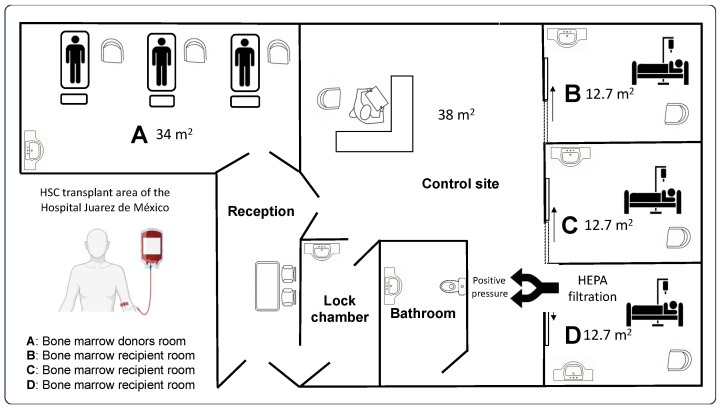
Aerial map of the HSC transplant area at HJM. (**A**) Donor room. (**B**–**D**) Recipient rooms.

The HSC transplant area has the necessary infrastructure for patient care according to the recommendations reported by Inkster et al. (2022), such as air filtration and hourly air changes (ACH) (room D only), room sealing with sanitary finishing, and epoxy-coated floors, among others [25]. Figure 1 shows the aerial-view map of the HSC transplant area at HJM, and Table 1 shows the infrastructure characteristics per room in the HSC transplant area.

### 2.2. Determination of Air Quality Parameters (PM, Temperature, and Relative Humidity)

The PM concentrations per m^3^ were measured annually (bimonthly) in the four rooms of the transplant area (A, B, C, and D). For this purpose, an ExTech VPC300 optical particle counter (OPS) (FLIR Commercial Systems Inc., Wilsonville, OR, USA), previously calibrated inside a type II laminar flow hood, was used. The device was configured to quantify airborne PM of 1.0, 2.5, and 10.0 μm during a sampling interval of 20 s according to the manufacturer’s conditions (per triplicate). The analysis conditions were implemented identically as in the quantification of the bacterial and fungal aerobiomes (in terms of floor height and time). Finally, the temperature (°C) and relative humidity (RH) (%) were simultaneously recorded (per triplicate) with a calibrated thermohygrometer.

### 2.3. Annual Analysis of the Aerobiome in the HSC Transplant Rooms at HJM

The quantification of the aerobiome in the HSC transplant rooms at HJM was carried out for one year, namely “January to December 2023” (bimonthly), at a fixed time (10:00 a.m.), considering the greatest influx of health personnel. For the quantification of the airborne microbiological load of fungi and Gram-negative and Gram-positive bacteria, the collision method was used with the Air Ideal 3P sampler (bioMérieux, Lyon, France), according to the manufacturer’s conditions, using a set of solid culture media for each room (A, B, C, and D). These were potato dextrose agar (PDA; plus chloramphenicol (30 μg/mL) and streptomycin (30 μg/mL)), Mannitol Salt a, and MacConkey agar, for the presumptive identification of fungi, Gram-positive bacteria, and Gram-negative bacteria, respectively.

For this purpose, the air sampler was programmed for 1000 L air sample aspiration volumes, equivalent to 1 m^3^, as follows. With the doors closed, the air sampler was placed 1.5 m above ground level in the central part of each room and switched on, and the operator immediately left the sampling point. After sampling, the culture media were immediately enclosed and incubated aerobically at 37 °C for 48 h and 28 °C for 72 h, as the growing conditions for bacteria and fungi, respectively. Bacterial and fungal colonies were counted and reported as CFU/m^3^ of air sampled; finally, all colonies were isolated on the same culture media to obtain pure cultures for identification as follows.

### 2.4. Bacterial Aerobiome Identification by Mass Spectrometry MALDI-TOF

Only pure bacterial isolates were identified by the direct analysis of whole bacterial cells using matrix-assisted laser desorption/ionization-time of flight mass spectrometry (MALDI-TOF MS). For this purpose, all bacterial strains were streaked in LB agar and incubated overnight at 37 °C, and single colonies were subjected to identification by using a Bruker MALDI Biotyper (Bruker Daltonik, Bremen, Germany), according to the manufacturer’s instructions. The criterion found to best match with the identification protocol was bacterial strains with score values above 2.0 (down to 3) for high-confidence identification. With the taxonomic assignment information of the isolates, plots of the relative abundance versus taxonomic diversity and seasonal heat maps of the taxonomic diversity (per room) versus bimonthly period of analysis were constructed.

### 2.5. Macro- and Microscopic Identification of the Fungal Aerobiome

Representative colonies of the aerial fungal load were chosen for axenic cultures on PDA agar. These cultures were incubated aerobically for 7 days at 28 °C for the description of the macro- and microscopic morphology using the method reported by Johnson and Borman (2010) [26]. To describe the macroscopic morphology, aspects such as the texture, colonial surface, color of the obverse/reverse, and presence of diffusible pigments were identified. The microscopic identification at 1000× total magnification of the mycelium and typical and asexual reproductive structures (hypha, conidiophore, microconidium, dicthyoconidium, vesicle, phialide, and metula) was performed using lactophenol cotton blue.

### 2.6. Molecular Typing of Gram-Negative Bacteria (ESKAPE) by ERIC-PCR

Gram-negative bacteria (only ESKAPE bacteria) were subjected to molecular typing by ERIC-PCR, by using the primers ERIC1R (5′-ATGTAAGCTCCTGGGGGGATTCAC-3′) and ERIC2 (5′-AAGTAAGTGACTGGGGGGTGAGC-3′), according to Versalovic et al., 1991 [27]. The total reaction volume was 50 μL and consisted of 1× PCR buffer, 20 nM MgCl_2_, 25 mM dNTPs, 100 pM of each primer, *Taq* DNA polymerase (2 U), and 300 ng of genomic DNA. The cycling conditions were as follows: pre-denaturation at 95 °C for 7 s, denaturation at 90 °C for 30 s, annealing at 58 °C for 1 min, and extension at 65 °C for 8 min, with a final extension at 68 °C for 16 min at the end for 30 cycles. Genetic profiles were run in 1 X TBE buffer, pH 8.3, and separated in horizontal electrophoresis in 1.5% agarose gels, visualized, photographed under UV illumination, and analyzed by intra-gel pattern comparison. To confirm the reproducibility of the ERIC-PCR assays, these were performed three times. The Tenover criteria were used to establish the clonal relationships between isolates with the same genus and species [28]. Finally, the graphical relationships were analyzed through a distance matrix by using a linear semilogarithmic method. The dendrograms were constructed using the UPGMA algorithm, with the Dice similarity index. Genomic similarity was confirmed with a bootstrap test of 1000 repetitions using the Past4 program (Version 4.09).

### 2.7. Statistical Analysis and Vester Matrix Construction

Significant differences between the variables analyzed were evaluated by using ANOVA and Tukey’s post hoc test for the concentrations of 1.0, 2.5, and 10.0 mg/m^3^; the temperature and relative humidity (RH); and the bacterial and fungal microbiological loads. Significant differences between the variables were established when the *p*-value was <0.05. Additionally, the SPSS v.27.0.1.0 and XLSTAT 2023 statistical software programs were used for the analysis and graphical representation. Finally, a Vester matrix was constructed to classify microbiological problems in the HSC transplant area according to their degree of causality. For this purpose, 24 situations were identified that could impact various outcomes of microbiological contamination. Causality was assessed individually and as a group by various health professionals, including 4 microbiologists, 4 oncologists, 4 hematologists, 4 epidemiologists, 4 nurses, and 4 cleaning assistants. With the results of causality (0, 1, 2, and 3), those that occurred most frequently were considered to have causality closest to reality and were transferred to the matrix to be categorized as active, passive, critical, or indifferent problems [29].

## 3. Results

### 3.1. HEPA Pressure and Positive Filtration Impacts the Standard Air Quality Parameters

**Figure 2:**
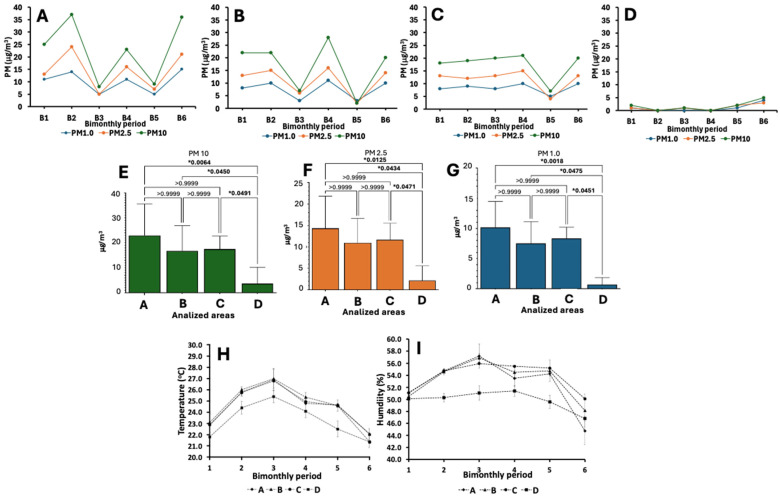
Standard air quality parameters of the HSC transplant rooms. PM10, PM2.5, and PM1.0 μm quantification (**A**–**D**) and Tukey’s post hoc and ANOVA tests for the comparison of the PM levels between rooms (**E**–**G**). Temperature (°C) and relative humidity (RH%) in the HSC transplant rooms (**H**,**I**). * Statistically significant (*p* ≤ 0.05).

To determine the impact of HEPA filtration and positive pressure in the transplant room, the annual quantification (mg/m^3^) of particulate matter (PM1.0, 2.5, and 10 μm) was performed. The results revealed that, in those rooms where HEPA filtration was not available (rooms A, B, and C), the maximum PM10, PM2.5, and PM1.0 levels of 37, 24, and 15 μg/m^3^, respectively, were detected. The minimum PM levels were at magnitudes of 3, 2, and 3 μg/m^3^ for PM10, PM2.5, and PM1.0, respectively. The ANOVA and Tukey’s post hoc analysis demonstrated significant differences for all particles analyzed between donor room A and recipient rooms B and C versus recipient room D (Figure 2).

Figure 2A–G show the results of the PM10, PM2.5, and PM1.0 μm quantification from the HSC transplant rooms at HJM and the Tukey’s post hoc analysis and ANOVA for the statistical comparison of the PM levels between the rooms. Simultaneously with these determinations, measurements of the ambient temperature and RH were performed. The results revealed average maximum temperature and RH values of 27 ± 0.4 °C and 57.2 ± 0.2%, respectively. Conversely, the minimum values of these same parameters were between 21.8 ± 0.3 °C and 44.8 ± 0.1%. Interestingly, these maximum and minimum values were directly related to the seasonality for warm months and cold months (B3 “May–June”) and (B4 “July–August”); however, regarding room D, it was the room with the lowest temperature and RH values during the study period. Figure 2H,I show the results of the temporal analysis of the temperature and RH in the HSC transplant area at HJM.

### 3.2. Seasonal Behavior of Aerobiome Load

The seasonal analysis of the cultivable bacterial and fungal aerobiomes of the HSC transplant rooms showed the maximum microbiological loads with peaks in the months of spring (May/June) and summer (July/August), with average viable counts of 1.0 × 10^2^ ± 22.6, 2.2 × 10^2^ ± 22.6, and 72.5 ± 38.9 CFU/m^3^ for rooms A (donors), B, and C (recipients). This seasonal behavior of the loads was not detected in room D (for recipients), which had HEPA filtration and positive pressure.

**Figure 3:**
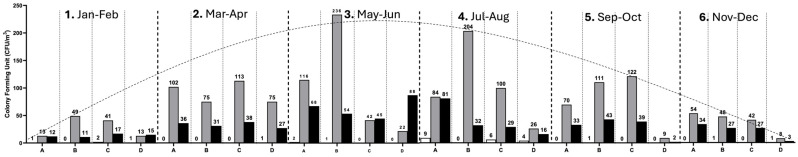
Seasonal analysis (bimonthly) of bacterial (Gram-positive/-negative) and fungal aerobiome microbiological loads analyzed in the HSC transplant area at HJM from January to December 2023. White bar: Gram-negative bacteria, gray bar: Gram-positive bacteria, and black bar: fungi.

The lowest microbiological loads were identified in the two-month periods at the extremes of the year, in winter (January/April) and autumn (October/December). The highest peak bacterial loads were represented by Gram-positive bacteria, followed by fungi and Gram-negative bacteria. Figure 3 shows the seasonal analysis (bimonthly) of the aerobiome microbiological loads that were analyzed in the HSC transplant rooms at HJM from January to December 2023.

### 3.3. Significant Differences in Bacterial Aerobiome Load Between Rooms

Because the recipient rooms (B, C, and D) and the donor room (D) presented microbiological loads with seasonal behavior (Figure 3), an ANOVA and Tukey’s post hoc test were performed to determine the existence of significant differences in bacterial microbiological contamination between the rooms. The results revealed the presence of significant differences between rooms B and D for receivers (*p =* 0.0197).

**Figure 4:**
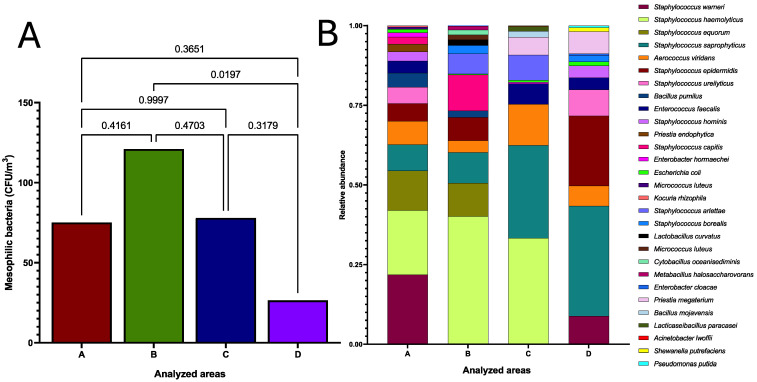
(**A**) ANOVA and Tukey’s post hoc test for the statistical comparison of the culturable bacterial aerobiome between transplant area rooms. (**B**) Bacterial identification findings (relative abundance and taxonomic diversity) by mass spectrometry MALDI-TOF of the culturable bacterial aerobiome (per room) of the HSC transplant area at HJM.

No differences between rooms A, B, and C were identified. In microbiological terms, it was observed that the degree of bacterial contamination was similar between rooms without HEPA filtration and positive pressure. Figure 4A shows the results of the ANOVA and Tukey’s post hoc test for the statistical comparison of the bacterial aerobiome, and Figure 4B summarizes the findings of the bacterial identification by mass spectrometry of the culturable aerobiome in the HSC transplantation area at HJM.

### 3.4. Clinically Important Taxonomic Diversity of Bacterial Aerobiome

To elucidate the taxonomic diversity and relative abundance (%) of the culturable bacterial aerobiome in the HSC transplant area, the mass spectrometry identification of all isolates per m^3^ from the four rooms (A, B, C, and D) was performed during the study period (Figure 4B). The results revealed the significant diversity of the clinically relevant bacterial genera and species in the four rooms analyzed (A = 9 genus and 16 species, B = 9 genus and 15 species, C = 9 genus and 11 species, and D = 8 genus and 13 species).

The aerobiome was mainly represented by Gram-positive bacteria, where the genus *Staphylococcus* spp. showed the highest relative abundance, with *S. warneri*, *S. haemolyticus*, *S. saprophyticus*, and *S. epidermidis* species being the most prevalent. Within this bacterial group (Gram-positive), *Enterococcus faecalis*, a bacterium belonging to the ESKAPE group, was identified. Alternatively, four bacterial genera of Gram-negative bacteria were identified, where three of them also belonged to the ESKAPE group (*Escherichia coli*, *Enterobacter cloacae*, and *E. hormaechei*).

Regarding the differences in the taxonomic diversity and relative abundance of the aerobiome between areas, it was identified that *S. haemolyticus* was present with abundances of 0.20, 0.40, and 0.33 in rooms A, B, and C, respectively, being rooms without HEPA filtration and positive pressure. In contrast, the relative abundance of *S. saprophyticus* increased gradually in each of the areas (A = 0.082, B = 0.096, C = 0.291), being higher in the room with HEPA filtration and positive pressure (D = 0.345).

The rest of the bacterial genera and species present in the aerobiome showed heterogeneous relative abundances in each of the rooms analyzed. Finally, *S. saprophyticus*, *Aerococcus viridans*, and *E. coli* (ESKAPE bacteria) were present in all four rooms, regardless of the type of use (for donors or recipients). Figure 4B summarizes the bacterial identification findings by mass spectrometry (MALDI-TOF MS) of the culturable aerobiome in the HSC transplant area (donor and recipient rooms) at HJM.

### 3.5. Seasonal Transition of the Bacterial Aerobiome in the HSC Transplant Area

**Figure 5:**
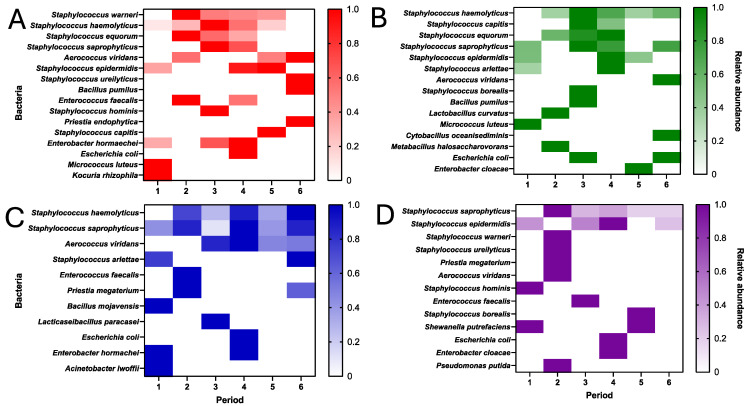
Heat maps of the seasonal behavior of the culturable bacterial aerobiome of the HSC transplant area at HJM during January to December 2023 (distributed according to bimonthly analysis). (**A**) Donor room, (**B**–**D**) recipient rooms.

As part of the analysis of the bacterial aerobiome, a longitudinal follow-up study of the behavior of the bacterial aerobiome per m^3^ of the HSC transplant area during the study period was carried out. For this purpose, heat maps were created of each of the rooms analyzed, using the information on the relative abundance and taxonomic diversity per m^3^ identified by mass spectrometry. The results revealed that species of the genus *Staphylococcus* showed a reduction in relative abundance during the bimonthly course of the study period. Species such as *S. warneri*, *S. haemolitycus*, *S. equorum*, and *S. saprophyticus* showed this behavior in the donor (A) and recipient (B and D) rooms (Figure 5).

Regarding room C, no seasonal transition (upward or downward) of this bacterial genus was observed. In the case of *Aerococcus viridans*, in addition to being identified in all four rooms, only in receiving room C did it show an increase in relative abundance during the study period.

Two members of the ESKAPE group (*E. faecalis* and *E. coli*) were detected in a timely manner from the second to the fourth bimester analyzed, without any change in relative abundance; only *E. coli* was detected at two different times (bimesters 3 and 6) in room B for recipients. Interestingly, the ESKAPE member *Enterobacter hormaechei* was detected in the donor room (A), with an increase in relative abundance towards the warmer months of the year (bimesters 1, 3, and 4).

Figure 5 shows the heat maps of the seasonal behavior of the bacterial aerobiome of the HSC transplant area at HJM during January to December 2023 (distributed according to bimonthly analysis).

### 3.6. No Significant Difference in Fungal Aerobiome Between Rooms

Regarding the fungal aerobiome loads, the ANOVA and Tukey’s post hoc test revealed the absence of significant differences between the microbiological loads per m^3^ of the aerobiome between the areas analyzed (*p* > 0.05). In microbiological terms, the degree of fungal contamination per m^3^ was similar between the areas, regardless of the room type (with or without HEPA filtration and positive pressure).

**Figure 6:**
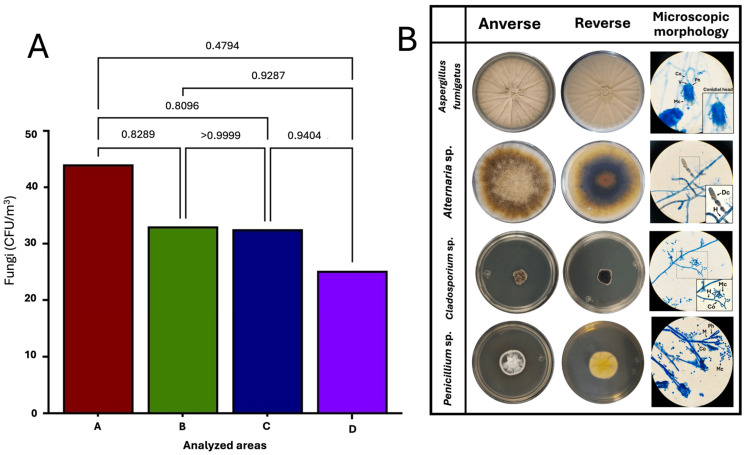
Fungal aerobiome of the HSC transplant rooms. (**A**) ANOVA and Tukey’s post hoc test for fungal aerobiome. (**B**) Macro- and microscopic identification of fungi in the aerobiome of the HSC transplant area at HJM. The colonial morphology (front and back): *Alternaria* sp., *Cladosporium* sp., *Aspergillus fumigatus*, and *Penicillium* spp. Sexual reproductive structures at 1000× total magnification. H = Hypha, Co = Conidiophore, Mc = Microconidium, Dc = Dicthyoconidium, V = Vesicle, Ph = Phialide, M = Metula.

Figure 6A shows the results of the ANOVA and Tukey’s post hoc test for the statistical comparison of the fungal aerobiome, and Figure 6B shows representative microphotographs of the macro- and microscopic identification findings for the filamentous fungal genera of the culturable aerobiome in the HSC transplant area at HJM.

### 3.7. Fungal Aerobiome Reveals Filamentous Fungi Involved in Nosocomial Fungemia

The macro- and microscopic analysis of the most frequent morphotypes of the filamentous fungi that were isolated in the rooms of the HSC transplant ward revealed the presence of clinically important genera involved in cases of fungemia and/or invasive mycosis in transplant patients, such as *Aspergillus fumigatus*, *Alternaria* spp., *Cladosporium* spp., and *Penicillium* spp. This was due to the observation of characteristic asexual reproductive structures of the isolates. The four fungal genera were homogeneously distributed in the four rooms. Figure 5B shows the morphological identification of filamentous fungi from the HSC transplant area at HJM during January to December 2023.

### 3.8. Molecular Typing of ESKAPE Members in the Aerobiome by ERIC-PCR

The profiles of the intergenic products obtained by end point-PCR for *E. cloacae* and *E. hormaechei* isolated from the aerobiome revealed that the sizes of the amplicons ranged from slightly more than ≈280 bp to about ≈1600 bp. The intergenic region diversity (six different amplicons) showed that the seven isolates of *E. hormaechei* (*n* = 4) and *E. cloacae* (*n* = 3) were grouped into two unique clonal groups. In the case of the seven isolates of *E. coli* from the aerobiome, it revealed that the sizes of the amplicons ranged from slightly more than ≈100 bp to about ≈1400 bp and were grouped into a single clonal group.

**Figure 7:**
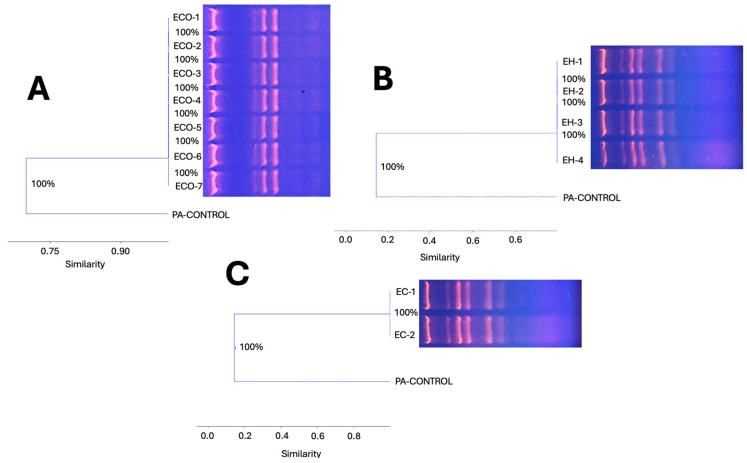
Genomic diversity (clonal dispersion) by dendrogram construction of ESKAPE bacteria detected in aerobiome in HSC transplant rooms of the Hospital Juárez de México. *Escherichia coli* (**A**), *Enterobacter hormaechei* (**B**), and *E. cloaceae* (**C**). PA (*Pseudomonas aeruginosa* as internal control).

Therefore, it is concluded that three clonal groups were identified, distributed across three Gram-negative bacterial genera from the ESKAPE group. The spatial distribution of these ESKAPE group clones (by room in the HSC transplant area) is shown in the heat maps in Figure 5. In Figure 7, the clonal dispersion dendrograms for *E. coli*, *E. hormaechei*, and *E. cloaceae* in the HSC transplant rooms at HJM are shown.

### 3.9. Vester Matrix Construction for Microbiological Situations in the Transplant Area

**Figure 8:**
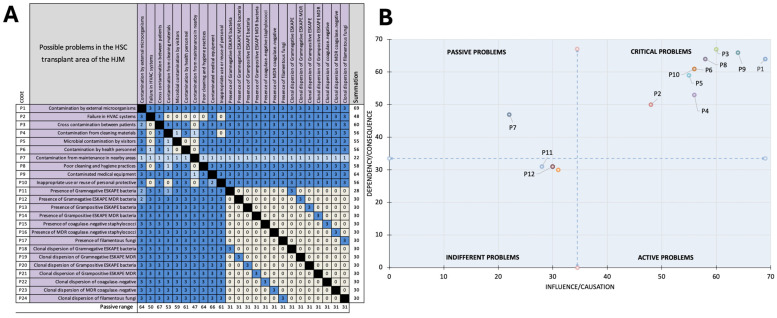
Microbiological risk analysis in the HSC transplant area at HJM. (**A**) Vester matrix of 24 controversial situations of potential microbiological contamination. (**B**) Identification and classification of problems according to the influence and dependency on the identified problems.

A Vester matrix was constructed to classify different situations according to their degree of causality and determine which ones resulted in microbiological problems in the HSC transplant area at HJM. Twenty-four main controversial situations were identified (Figure 8A). Additionally, the degree of causality of the microbiological findings was analyzed by the type of pathogen, whether it was multidrug-resistant (MDR) or non-MDR, and the clonal dispersion of the microorganisms. The results revealed that, of the controversial situations (24/100%), nine critical problems (37.5%), one passive problem (4.1%), zero active problems (0%), and fourteen indifferent problems (58.3%) were identified, with contamination by external microorganisms (P1) being the most relevant critical problem, followed by the contamination of medical equipment (P9) and cross-contamination between patients (P3). In contrast, bacterial (including MDR) and fungal microbiological problems (P11-P24) were grouped as indifferent problems; however, they were closely related to the critical problems as they were the result of the indifferent problems. Figure 8A shows the Vester matrix with the 24 controversial situations that could impact the microbiological characteristics of the HSC transplant area at HJM, and Figure 8B shows the spatial distribution of the passive, critical, indifferent, and active problems.

## 4. Discussion

Opportunistic infections in oncology patients are a challenge in hospital settings, as they pose significant risks to the patients who suffer from them, with the depletion of the immune system being the main factor of susceptibility. Even when the HSC transplantation procedure is successful, poor air quality and microbiological control can exacerbate the inherent risk of this medical procedure, allowing the spread of opportunistic pathogens of bacterial and/or fungal origin and consequently the development of infections. These microorganisms, which, under normal conditions, would not cause disease, can cause life-threatening infections in immunocompromised patients.

Therefore, it is crucial to implement strict airborne microbiological surveillance and control measures in the areas where patients are kept in order to minimize exposure to airborne contamination and consequently reduce the incidence of opportunistic infections and thus increase the chances of recovery.

Subsequently, the objective of the present study was the seasonal characterization of the aerobiome in the HSC transplant area at HJM, to relate the microbiological findings to the infrastructure of this area and to possible weaknesses in the operability of the health personnel, which could have an impact on the characteristics of the aerobiome. As observed in Figure 1 and Table 1, the area coded as “room D” was the only room with HEPA filtration and positive pressure infrastructure. These features showed a positive impact in this area, with the lowest levels of PM > 1.0, 2.5, and 10 μm (Figure 2A–G). High-efficiency HEPA filtration has been recognized as one of the best alternatives in reducing PM, including infectious bioaerosols, in various environments, such as domestic environments and areas in and around schools [30,31,32]. Concerns about the negative impact of PM and the development of chronic degenerative diseases and the need for its reduction in the hospital environment have already been described [33,34].

An important aspect to consider is that HEPA filtration systems are designed to reduce the PM load (including bacteria, spores, etc.); however, it is not a system that promotes zero microbiological contamination rates. A relevant finding is shown in Figure 3, where the seasonal influence on the fluctuations in the microbial load can be identified; this may indicate that certain periods have the most favorable conditions for microbial growth, such as the temperature and RH, which showed upward behavior in the hot months (Figure 2H,I). These results provide insights into the dynamics of microbial loads, highlighting the importance of more rigorous monitoring and control, such as the immediate implementation of improved hygiene practices and environmental control in those months where the conditions are most favorable for microbial growth.

This information can be crucial as it confirms that filtration systems act as barriers to microbiological contamination. This can be seen in Figure 4A, where it is shown that, even though significantly different rates (*p =* 0.0197) of airborne contamination were detected in the controlled room (D) compared to uncontrolled areas, it could be detected and quantified during the study period. In contrast, the findings for the fungal load showed no significant differences between these same areas, even with the presence of HEPA filtration and positive pressure in room D (Figure 6A), suggesting the homogeneous distribution of these microorganisms in the hospital environment, regardless of seasonality and the influence that the temperature and RH may have on their presence and abundance, as fungal spores are known to be resistant to factors such as the temperature, humidity, water availability, and others. The influence of seasonality on the behavior of the bacterial and fungal loads (Figure 3 and Figure 5) has already been reported in a previous work investigating the aerobiome in hospital environments. Nuñez and García (2023) characterized the hospital aerobiome to determine seasonal trends and the impact on window opening [35]. This work highlighted the complex taxonomic composition and relative abundance of the bacterial load in the autumn and summer months, where it is speculated that factors such as the ambient temperature may influence the changes in the taxonomic diversity and relative abundance of the aerobiome.

Regarding aerobiome characterization, while the taxonomic diversity analysis revealed bacteria predominantly from the coagulase-negative staphylococci (Figure 4B), which are considered to be environmental bacteria, seven of the ten species identified have been reported as opportunistic pathogens in patients with HSC transplants (Table 2).

We speculate that the heterogeneity in the presence and absence of species such as *S. warneri*, *S. equorum*, and *S. epidermidis* in the analyzed areas arises from multifactorial contamination events that could be influenced by various causes; however, the predominance of *S. epidermidis* in “room D” provides indications of contamination by bacteria from entirely biological (skin) sources. In contrast, the ESKAPE group of bacteria has been recognized as one of the main microbiological problems in hospital centers, mainly where critical patients are cared for.

During the COVID-19 pandemic, the ESKAPE group was identified as the group of microorganisms that led to the worsening of patients, as they generated co-infections with the SARS-CoV-2 virus and formed some of the bacterial contaminants on surfaces and/or medical devices [14,21,36]. In a worldwide report by the Antimicrobial Resistance Collaborators, a systematic analysis was provided for the 2019 Global Burden of Disease Study, where global mortality was associated with 33 bacterial pathogens, with the ESKAPE group being the main one, ranking among the top ten infectious bacterial agents causing death in susceptible patients. In particular, *E. coli*, the genus *Enterobacter*, and *E. faecalis* were ranked second, seventh, and ninth, respectively [37]. The microbiological findings of these three members of the ESKAPE bacteria, together with the clonal spread of Gram-negative bacilli in the rooms of HSC recipients, could reflect one of the most important exogenous contamination health problems in transplant areas in this work (Figure 7 and Table 2).

**Table 2:** Summary of opportunistic infections in patients with HSC transplantation reported in the scientific literature and compared with the findings of the present work.

Pathogen	Genus	Species	Infections in HSC Transplant Patients	References
Gram-Positive	*Staphylococcus*	*S. warneri*	Bacteremia, oral disbiosis, oral mucositis	[38,39,40]
		*S. haemolyticus*	Bacteremia, meningitis, oral mucositis	[38,39,41]
		*S. equorum*	Surgical site in liver transplant patients	[42]
		*S. saprophyticus*	Bacteremia	[43]
		*S. epidermidis*	Bacteremia	[18]
		*S. ureilyticus*	Non-reported	NA *
		*S. hominis*	Bacteremia	[44]
		*S. capitis*	Bacteremia	[18]
		*S. arlettae*	Non-reported	NA *
		*S. borealis*	Non-reported	NA *
	*Enterococcus*	*E. faecalis*	Bacteremia, enteric mucositis	[45,46,47]
	*Micrococcus*	*M. luteus*	Catheter-related septic shock	[48]
	*Aerococcus*	*A. viridans*	Non-reported	NA *
	*Kocuria*	*K. rhizophila*	Catheter-related bacteremia	[49]
	*Bacillus*	*B. pumilus*	Non-reported	NA *
		*B. mojavensis*		
	*Lactobacillus*	*L. curvatus*		
	*Lacticaseibacillus*	*L. paracasei*		
	*Cytobacillus*	*C. oceanisediminis*		
	*Metabacillus*	*M. halosaccharovorans*		
	*Priestia*	*P. endophytica*		
		*P. megaterium*		
Gram-Negative	*Enterobacter*	*E. hormaechei*	Rectal and intestinal colonization	[50,51]
		*E. cloacae*	Gut and urinary tract infections, bacteremia	[52,53,54,55]
	*Escherichia*	*E. coli*	Bacteremia, gastrointestinal colonization	[56,57,58]
	*Acinetobacter*	*A. Iwoffii*	Bacteremia, colitis, pneumonia	[59,60,61]
	*Shewanella*	*S. putrefaciens*	Bacteremia	[62,63]
	*Pseudomonas*	*P. putida*	Bacteremia	[61,64]
Fungi	*Alternaria*	*Alternaria* spp.	Cutaneous phaeohyphomycosis, cutaneous alternariosis, fungal infection (brain, sinus, and skin)	[65,66,67]
	*Cladosporium*	*Cladosporium* spp.	Invasive mold infections	[68,69]
	*Aspergillus*	*A. fumigatus*	Rhino-cerebral, cutaneous and pulmonary aspergillosis, disseminated, and gastrointestinal	[70,71]
	*Penicillium*	*Penicillium* spp.	Invasive fungal infection, pulmonary fungal infection	[72,73]

* NA: Non-applicable.

The clonal spread of the ESKAPE group bacteria has already been reported at the HJM in other critical patient care areas, the most important being the ICU for COVID-19 patients. In this context, Loyola-Cruz et al. (2023) and Durán-Manuel et al. (2021) identified the clonal spread of *Acinetobacter baumannii* MDR and *Pseudomonas aeruginosa* MDR/XDR in patients with ventilator-associated pneumonia (VAP) and as microbiological contamination in medical devices in the ICU, and this problem was exposed with an emphasis on the importance of adopting good practices in patient management and cleaning and disinfection protocols [14,74]. As shown in Figure 4B, even though the frequency of the airborne isolation of these microorganisms in the transplant area was low, their isolation has clinical relevance due to the possibility of their clonal spread on surfaces and medical devices and in patients. Finally, the mycological findings and their clinical relevance are summarized in Table 2. In recent years, filamentous fungi such as *Alternaria* spp. and *Cladosporium* spp. have been considered as emerging pathogens in transplant patients, causing life-threatening skin and invasive infections [66,68].

The fungus *A. fumigatus* is particularly relevant due to its high production of enzymes with proteolytic activity, which gives it an enormous capacity to cause a variety of serious infections, such as pulmonary mycosis to disseminated infections [75]. This is why the early detection and treatment of fungal infections is critical due to the high mortality associated with them. It is important to mention that, during the study period, eleven HSC transplants were performed in room D, where two cases of post-transplant opportunistic mycoses were detected (18%). These cases were represented by two male patients aged 17 and 53 years, with mucositis and pneumonia caused by *Candida* spp. and *Aspergillus fumigatus*, respectively, with ALL and multiple myeloma being the oncohematological pathologies associated with these two cases. The above discussion highlights two needs: infrastructure to control these parameters in all areas of care and mainly changes that impact the management of critically ill patients by healthcare personnel. For this reason, a risk matrix was generated, which detected critical problems that could directly influence microbiological contamination in these areas, considering various controversial situations, such as the relaxation of entry protocols to controlled areas, the inappropriate use or reuse of personal protection equipment (PPE), failures in the ventilation system, contamination by visitors, etc. (Figure 8). This tool facilitated an understanding of the controversial situations in this area in a simple and practical way (Figure 8A). We identified that, even if an area has positive pressure infrastructure and other microbiological control features, as shown in Table 1, these will be of low impact if the active problems (P1-P6 and P8-P10) are not immediately addressed.

This is in accordance with the results shown in the problem distribution map in Figure 8B. It is important to mention that the indifferent problems may be underestimated under the definition that they are those that are not exclusively related to the critical problems, as they may be the outcomes of the situations that give rise to active problems. Therefore, the fundamental purpose of this type of research is to encourage adherence to all activities that could lead to exogenous microbiological contamination in controlled areas, rather than merely proposing that positive pressure and filtration infrastructure is the only way to ensure microbiologically “clean” critical areas. As mentioned, transplantation is a complex medical procedure; however, these medical efforts can be hampered by bacterial and fungal infections, so the air in the rooms where these procedures are performed should be subject to surveillance. Alternatively, the seasonal variability in the degree of microbiological contamination highlights the need to implement more rigorous environmental control strategies during periods of increased risk.

## 5. Conclusions

This study shows the importance of monitoring and controlling the aerobiome in HSC transplant rooms to minimize the risk of nosocomial infections, where the implementation of measures such as HEPA filtration and the maintenance of positive pressure in critical areas is essential to improve clinical outcomes and reduce the morbidity and mortality associated with infections in immunocompromised patients.

## Figures and Tables

**Table 1:** Infrastructure by room (donors and recipients) in the HSC transplant rooms at HJM.

Characteristic	Unit	Donor Room	Recipient Rooms		
		A	B	C	D
Dimensions	m^2^	34	12.7	12.7	12.7
Positive pressure	Pa	No	No	No	Yes
HEPA filtration	0.3 mm	No	No	No	Yes
Air changes	AC/h	0	0	0	10
Sanitary sealing	Non applicable	No	Yes	Yes	Yes
Epoxy floor coating		Yes	Yes	Yes	Yes
Shared bathroom between patients		No	Yes	Yes	Yes
Single washbasin		Yes	Yes	Yes	Yes

## Data Availability

Bello-López, Juan Manuel (2024), “Seasonal Characterization of the Aerobiome in Hematopoietic Stem Cells Transplant Rooms: Potential Risk for Immunosuppressed Patients”, Mendeley Data, V1, http://doi.org/10.17632/58vc8fcyd9.1.
